# Delays in the Reticulospinal System Are Associated With a Reduced Capacity to Learn a Simulated Feeding Task in Older Adults

**DOI:** 10.3389/fncir.2021.681706

**Published:** 2022-01-27

**Authors:** Vishvak Rangarajan, Joseph J. Schreiber, Beatriz Barragan, Sydney Y. Schaefer, Claire F. Honeycutt

**Affiliations:** School of Biological and Health Systems Engineering, Arizona State University, Tempe, AZ, United States

**Keywords:** aging, learning, reticulospinal, skill acquisition, startle, brainstem, reticular formation, motor learning

## Abstract

Learning declines with age. Recent evidence indicates that the brainstem may play an important role in learning and motor skill acquisition. Our objective was to determine if delays in the reticular formation, measured via the startle reflex, correspond to age-related deficits in learning and retention. We hypothesized that delays in the startle reflex would be linearly correlated to learning and retention deficits in older adults. To determine if associations were unique to the reticulospinal system, we also evaluated corticospinal contributions with transcranial magnetic stimulation. Our results showed a linear relationship between startle onset latency and percent learning and retention but no relationship between active or passive motor-evoked potential onsets or peak-to-peak amplitude. These results lay the foundation for further study to evaluate if (1) the reticular formation is a subcortical facilitator of skill acquisition and (2) processing delays in the reticular formation contribute to age-related learning deficits.

## Introduction

Older adults learn at a slower rate and to a lesser extent than younger adults (Gunning-Dixon et al., 2002; Seidler, 2007; Roig et al., 2014; King, 2016). This population also demonstrates a decreased ability to transfer learning from one skill to another (Walter et al., 2019). This is particularly problematic for older adults who are at high risk for neurological disease (e.g., stroke) whose treatment requires significant amounts of therapy to return to daily life. As therapy is the process of skill re-learning, a poor capacity to acquire new skills places older adults at a significant disadvantage. Despite its importance, the neural mechanisms underlying older adult’s poor learning capacity remain poorly understood.

The critical role of the cortex and corticospinal system in skill acquisition is well documented (Lawrence and Hopkins, 1976; Mengia et al., 1998; Kawai et al., 2015). However, recent work indicates that the role of these structures is altered throughout the course of learning. Initial learning of a task requires substantial input from the cortex; however, following intense training, rats can perform a sequence of precisely timed lever presses following bilateral motor cortex lesions (Kawai et al., 2015). A follow up study, also in rodents, shows a disengagement of M1 following long-term training (Hwang et al., 2019). This disengagement is not isolated to rats. Non-human primates show dexterous movement following large, gray-matter cortical lesion and expert songbirds can perform songs with appropriate frequency and amplitude modulations following bilateral forebrain cortical lesions (Andalman and Fee, 2009; Fee and Goldberg, 2011). Importantly, neither rats nor songbirds can learn new tasks or songs if cortical lesioning occurs before learning; this highlights a critical role for both cortical and subcortical systems during learning but also suggests that following learning subjects become increasingly reliant on subcortical systems for execution of skilled tasks.

While there are several subcortical structures that may contribute to this process (e.g., basal ganglia, red nucleus), human studies indicate that the brainstem, specifically the reticular formation, maybe a mediator of movement following skill acquisition. The startReact (SR) response, which numerous studies have used as a probe of the reticular formation (Thevathasan et al., 2011; Honeycutt et al., 2013; Nonnekes et al., 2014; Baker and Perez, 2017; van Lith et al., 2018; Choudhury et al., 2019; Sangari and Perez, 2019, 2020; Bartels et al., 2020; Maslovat et al., 2020), demonstrates changes with task expertise. StartReact is absent in novices or at the beginning of training but is present in experts or following training (Kirkpatrick et al., 2018; Bartels et al., 2020) suggesting that the reticular formation may be increasingly engaged as a task is learned.

To date, no one has evaluated the impact of age-related changes in the reticular formation on learning. The brainstem has known age-related deficits including cell shrinkage and death in rodents (Sabel and Stein, 1981) as well as volume loss in humans (Frackowiak et al., 2013). Correspondingly, the startle reflex has delays and deficits with age (Kofler et al., 2001; Tresch et al., 2014). The objective of this study was to determine if delays in the startle reflex, which is mediated by the caudal pontine and medullary medullary portions of the reticular formation (Davis and Gendelman, 1977; Davis et al., 1982; Koch et al., 1992; Yeomans and Frankland, 1996), correspond to age-related deficits in learning and retention of a simulated feeding task which has been developed and validated for quantifying age-related deficits in learning and retention. We hypothesized that delays in the startle reflex would be correlated to learning and retention deficits in older adults. We additionally evaluated corticospinal latencies and peak-to-peak amplitudes via transcranial magnetic stimulation (TMS) to determine if associations with learning & retention were unique the reticulospinal system.

## Methods

Twenty-eight healthy older adults (Age = 70 ± 7.7 years) participated. Inclusion criteria included: 50+ years old, a strong hand preference, no neurodegenerative disease, no diagnosed psychiatric condition, no prescribed hearing loss, no heart conditions, and no history of seizures. Consent was obtained via guidelines set by Arizona State University IRB protocols: STUDY00004214 and STUDY00002440. Subjects completed the experimental protocol over six testing sessions over 58 days. On session 1 (Day 1), subjects were consented, and clinical tests administered: grip strength testing via Dynamometer, tactile sensation (Semmes Weinstein), and hand dominance (Edinburgh Handedness Inventory). Dexterity was assessed with grooved pegboard assessment.

To evaluate motor learning, subjects were trained on a simulated feeding task. We have previously described the simulated feeding task, which has been shown to be feasible and efficacious in promoting motor learning in older adults (Schaefer et al., 2015; Walter et al., 2019; VanGilder et al., 2021). Briefly, subjects sat in front of a task board with a home cup with 30 raw kidney beans and three other cups placed in front of the subject at 450° and 135°. A spoon was placed 5 cm from the home cup by the subject’s non-dominant hand. Subjects were instructed to move two beans at a time from the home cup to the target cups in successive order starting with the one closest to their non-dominant hand. Subjects were timed from spoon pickup until the final beans were placed in the cup. Percent improvements and percent retention were the primary dependent variables and were calculated as follows:


Baseline⁢Trial⁢Time-Day⁢ 28⁢Final⁢Trial⁢TImeBaseline⁢Trial⁢Time× 100⁢and

Baseline⁢Trial⁢Time-Day⁢ 58⁢Final⁢Trial⁢TImeBaseline⁢Trial⁢Time×100.

During Session 1 (Day 1), subjects performed two trials (15 movements each) to assess baseline performance. Sessions 2, 3, and 4 were spaced 1 week apart (Days 7, 14, and 28). Subjects trained by completing 50 trials of the task (Total: 150 trials or 2,250 movements). Session 5 (Day 58) was completed 30 days after Session 4 and consisted of two trials to test retention. In Session 6 subjects’ startle and TMS onset latencies were evaluated. Session 6 was collected after all training and 1-month retention testing was completed.

### Startle Protocol

Subjects were seated in a chair directly in front of a startle horn which was placed 30 cm behind the subject. Electromyography (EMG) data were recorded at gain of 1,500 and frequency of 3,000 Hz by a 32-channel, 16-bit data acquisition system [NI USB-6363, National Instrumentation, Austin, TX, United States]. This system has a bandwidth of 10–1,000 Hz, an input impedance of 10 GΩ, and a common mode rejection ratio of 115 dB at 60 Hz. EMG bipolar electrodes (solid gel, Ag-AgCl surface electrode) were placed over Left and Right Sternocleidomastoid muscles (LSCM and RSCM). Subjects were randomly exposed to five loud startling acoustic stimuli of 122 dB. A decibel meter was used prior to each experiment to verify the sound intensity. The startling sound was generated by Siren Speaker TS-333S, 12 V DC/1,000 mA with a duration of 0.01 s and rise time of 0.002 s. Trials were delivered randomly no less than 5 s apart. Startle onset latency was the primary dependent variable. Onset latency was evaluated using a custom Matlab R2018a script. DC offset was removed and a 10-point moving average filter was applied to the rectified EMG signal. The custom script first marked the point where the measured data rose above the maximum background activity (500 ms) for at least 20 ms. All onsets were visually inspected by an experimenter blinded to trial type and motor learning performance of the subject.

### Transcranial Magnetic Stimulation Protocol

Twenty subjects also received TMS. EMG was collected over the right first dorsal interosseous muscle (FDI). Single pulse TMS was delivered using a Magstim 200 stimulator (Magstim, Whitland, Carmarthenshire, United Kingdom) with a figure-of-eight-coil (7 cm outer diameter of wings), and monophasic current waveform. To localize the FDI muscle representation within M1, participants were instructed to keep their right FDI abducted with a constant muscle contraction. An EEG cap was used to demarcate TMS coil position. The TMS coil was placed 33% of the way between the Cz reference and the left preauricular point, oriented 45° obliquely to the sagittal midline. After identifying the M1 FDI representation, the subject was asked to relax. Single pulse TMS were delivered until finding the resting motor threshold (RMT) for the right FDI, i.e., the minimum intensity to elicit 50 μV MEP in 5 out of 10 consecutive trials. TMS was delivered at 120% of RMT. Fifteen trials of both Active and Passive Motor Evoked Potentials (MEPs) were collected. The primary dependent variables for the TMS study was MEP onset latency and peak-to-peak amplitude and was statistically assessed like startle onset latency.

### Statistical Analyses

Linear regressions with 95% confidence intervals were performed with startle latency and active/passive MEP latencies and peak-to-peak amplitude as the independent variables with baseline performance, percent improvement, percent retention, age, grip strength, and probability of startle as the dependent variables.

## Results

**FIGURE 1:**
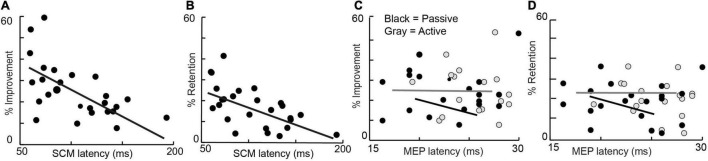
Percent improvement (left) and Percent retention (right) are depicted against average startle (SCM) onset latency, active MEP Latency, and passive MEP onset latency of all subjects.

Linear regressions demonstrated a relationship between startle onset latency and %improvement and %retention but not baseline performance, age, grip strength, or probability of startle (Figures 1A,B). Percent improvement (25.3 ± 12.4; *R*^2^ = 0.35, *P* < 0.001) and percent retention (17.6 ± 9.6; *R*^2^ = 0.38, *P* < 0.001) showed a relationship with startle onset latency. No other metrics were related to startle onset: baseline performance (*R*^2^ = 0.09, *P* = 0.13), age (*R*^2^ = 0.0045, *P* = 0.78), grip strength (*R*^2^ = 0.0052, *P* = 0.76), and probability of eliciting startle (0.71 ± 0.29; *R*^2^ = 0.0941, *P* = 0.19). More subjects were included in the startle group (*n* = 28) compared to the TMS group (*n* = 20); however, when only the 20 subjects that were common in both groups are considered, the primary metrics of percent improvement (*R*^2^ = 0.32; *P* = 0.008) and percent retention (*R*^2^ = 0.32; *P* = 0.01) still show a relationship with SCM onset latency.

Linear regressions did not demonstrate a relationship between active or passive MEP onset latencies and %learning and %retention, baseline performance, age, grip strength, and probability of eliciting startle (Figures 1C,D). Percent improvement (*R*^2^ = 0.01, *P* = 0.19), percent retention (*R*^2^ = 0.0014, *P* = 0.87), baseline performance (*R*^2^ = 0.0904, *P* = 0.87) age (*R*^2^ = 0.0626, *P* = 0.29), grip strength (*R*^2^ = 0.0518, *P* = 0.33), and probability of eliciting startle (*R*^2^ = 0.0103, *P* = 0.67) were not linearly related to active MEP latency. Percent improvement (*R*^2^ = 0.0133, *P* = 0.63), percent retention (*R*^2^ = 0.0438, *P* = 0.38), baseline performance (*R*^2^ = 0.016, *P* = 0.87), age (*R*^2^ = 0.021, *P* = 0.54), grip strength (*R*^2^ = 0.007, *P* = 0.71), and probability of eliciting startle (*R*^2^ = 0.038, *P* = 0.41) were not linearly related to passive MEP latency. Finally, peak-to-peak MEP amplitude was also not linearly related to percent improvement (*R*^2^ = 0.008, *P* = 0.69) or percent retention (*R*^2^ = 0.132, *P* = 0.11).

## Discussion

Our results showing a linear relationship with startle onset latency and percent learning and retention lay the foundation for further study to evaluate if (1) the reticular formation is an important subcortical facilitator of skill acquisition and (2) processing in this structure may contribute to age-related learning deficits. The lack of a relationship between MEP onset latency and amplitude and learning and retention indicates that while age-related deficits are correlated to delays in the reticulospinal system, they are not related to corticospinal delays.

### Role of the Reticular Formation in Motor Learning

There is growing evidence that following task acquisition, subcortical structures play an increasing role in movement execution. This study indicates that the brainstem, specifically the reticular formation, is an important subcortical facilitator of motor learning and retention of that learning. Our results also suggest that reticular formation deficits contribute to poor skill acquisition in older age. Aging influences several reticular-mediated functions such as sleep-wakefulness cycle (Hut and Van der Zee, 2011), auditory brainstem response (Backoff and Caspary, 1994), and saccadic eye movements (Wilson et al., 1993). There are also age-related anatomical changes including cell shrinkage and neuronal loss in the brainstem of rodents (Sabel and Stein, 1981) and volume loss in the brainstem of humans (Frackowiak et al., 2013). Taken together with the results presented strengthens the argument for the brainstem, in particular the reticular formation, to play an important role in age-related learning and retention deficits.

There is additional casual evidence that the brainstem may be contributing to learning deficits. One of the most sensitive cognitive measures for predicting learning deficits in older adults may be visuospatial function (Lingo VanGilder et al., 2019; Wang et al., 2020). There are close relationships between visual input receptive fields in the superior colliculus and the reticular formation (Philipp and Hoffmann, 2014). Further, visuomotor tasks where subjects must make fast motor corrections to visual inputs are also mediated via these structures (Kozak et al., 2019). This suggests, albeit indirectly, that one of the reasons visuospatial function predicts learning is due to age-related changes in the brainstem.

Finally, while we have interpreted our results to indicate that deficits in the reticulospinal system lead to a decreased capacity to learn and retain motor skills, an alternative interpretation is motor skill acquisition strengthens reticulospinal system inputs. Startle data were only collected at the conclusion of training – a limitation of this study. Future studies should evaluate pre- and post-training startle responses to gather evidence to support or refute this alternative hypothesis. Regardless, both interpretations highlight the importance of the reticulospinal system in motor skill acquisition.

### Cortico-Reticular Connections

The startle reflex is mediated by the reticular formation but is modulated by cortical inputs (Davis and Gendelman, 1977). Thus, delays in the startle reflex may correspond to age-related changes and atrophy in the cerebral cortex. We found no relationship between MEP latency and learning and retention, but this only indicates that conduction time of corticospinal tracts is not impacted while deficits in gray matter processing are likely still present in the cortex. Still, the cortex suppresses the startle reflex indicating an inhibitory role. If damaged, it would seem more likely to see an excitatory impact on the startle reflex as opposed to a delay. The scope of this manuscript does not allow full elucidation of the role the cortex plays in age-related changes to the startle reflex but does indicated further study is warranted.

### Implications for Motor Rehabilitation

Older adults are less responsive to therapy than younger patients (Dobkin et al., 2014). Startle may provide a non-invasive tool to predict the capacity of older adults to retain learning. This report shows that the onset latency of startle is related to learning deficits. A larger study could be conducted to determine how sensitive startle is to motor learning and if it could be used as a predictor. Startle is an easy and inexpensive behavioral measure to evaluate, which could be easily implemented in the clinic including rural and economically disadvantaged locations. *A priori* identification of patients who may be at risk of being minimally responsive to motor therapy allows clinicians to tailor the amounts and types of training to maximize skill learning.

## Data Availability Statement

The raw data supporting the conclusions of this article will be made available by the authors, without undue reservation.

## Ethics Statement

The studies involving human participants were reviewed and approved by Arizona State University IRB protocols: STUDY00004214 and STUDY00002440. The patients/participants provided their written informed consent to participate in this study.

## Author Contributions

CH contributed to the conception of the project, data analysis, manuscript preparation, and funded the project. VR and JS collected the data, performed the analyses, and assisted in manuscript preparation. BB assisted with data collection and analysis, and manuscript preparation. SS assisted with data collection, data analysis and manuscript preparation. All authors contributed to the article and approved the submitted version.

## Conflict of Interest

The authors declare that the research was conducted in the absence of any commercial or financial relationships that could be construed as a potential conflict of interest.

## Publisher’s Note

All claims expressed in this article are solely those of the authors and do not necessarily represent those of their affiliated organizations, or those of the publisher, the editors and the reviewers. Any product that may be evaluated in this article, or claim that may be made by its manufacturer, is not guaranteed or endorsed by the publisher.
